# Study and Analysis of Window Characteristics During Continuous Grain Drying

**DOI:** 10.3390/foods15091613

**Published:** 2026-05-06

**Authors:** Xing Jin, Wenfu Wu, Jiale Zhang, Feng Han, Yan Xu, Ruimin Li, Zhe Liu

**Affiliations:** College of Biological and Agricultural Engineering, Jilin University, Changchun 130022, China

**Keywords:** grain drying, drying window characteristics, equivalent accumulated temperature, actual accumulated temperature, simulation model

## Abstract

Grain drying is a pivotal post-harvest process that safeguards the storage safety and quality of grain. Conventional drying control strategies, however, predominantly rely on empirical operations and single-parameter monitoring. Although the concept of accumulated temperature has been applied in grain drying, few studies have systematically investigated the dynamic characteristics of drying accumulated temperature windows, resulting in a lack of quantitative and stable control criteria for the drying process. This study first defines the drying accumulated temperature window and further classifies it into three types: the equivalent window, actual window, and good window. On this basis, the window characteristics during continuous grain drying are systematically analyzed, accurate calculation methods for equivalent and actual accumulated temperature are established, and a feasible judgment criterion for the good window is proposed. A MATLAB 2022-based simulation model for continuous corn drying is constructed to verify the proposed methods. Experimental results show that three types of windows exhibit distinct dynamic response characteristics: the equivalent accumulated temperature responds instantaneously to changes in drying conditions, while the actual accumulated temperature has a time lag of one complete drying cycle. After the drying process stabilizes, the absolute difference between equivalent and actual accumulated temperature is controlled within 1500 °C·min. A drying process is identified to enter the good window state when the outlet moisture content stably maintains at 14.5 ± 0.5% for more than 3 h. The established simulation model demonstrates high prediction accuracy, with the mean relative errors of key indicators maintained at approximately 5%. This study clarifies the dynamic mechanism of accumulated temperature windows in continuous grain drying and provides a practical quantitative basis for the intelligent control and efficiency improvement of the grain-drying process.

## 1. Introduction

The concept of accumulated temperature was first proposed in 1735 by the French scientist Aolaier Delier, who defined it as the accumulation of daily average temperatures required for plants to complete their life cycles [1]. In 1923, Houghton and Yaglou introduced the concept of effective temperature and initiated relevant research on its application in biological processes [2]. Since its proposal, effective accumulated temperature has been widely applied in agricultural and biological research fields, including crop growth and development prediction [3,4,5], crop yield prediction [6,7,8], cultivation system optimization [9,10], and plant pest and disease control [11,12]. In recent years, this concept has been extended to the research of grain drying.

The grain-drying process shares similarities with plant growth and development in that drying high-moisture grain to a safe moisture content requires a certain amount of heat accumulation, which is defined as grain-drying accumulated temperature. Wu et al. [13,14] first proposed the application of the accumulated temperature concept in the grain-drying process, laying a theoretical foundation for its subsequent research and application. Liu et al. [15,16] developed a measurement method for equivalent accumulated temperature during drying and verified its applicability under different drying conditions. On this basis, Wu [17], Qi [18], Wang [19], and Jin [20] established various grain-drying control systems based on equivalent accumulated temperature, realizing the preliminary quantitative control of the drying process. Wu et al. [21] explored the drying characteristics of corn under different drying conditions and established a multi-parameter accumulated temperature regression model with a high fitting degree (*R*^2^ = 0.97) through controllable thin-layer hot-air drying experiments. Li et al. [22] developed a rice-drying model based on accumulated temperature and optimized the drying process parameters, which significantly improved drying efficiency and retained rice quality. Jin et al. [23,24] studied the correlation between accumulated temperature and rice quality and confirmed that the accumulated temperature required for rice to reach safe moisture content with high quality is relatively stable under specific drying conditions.

The application of the window concept in the grain storage field can be traced back to around 2014, where ventilation windows were mainly used for grain bin cooling and ventilation to regulate grain moisture and temperature, thereby delaying grain quality deterioration and inhibiting the growth of harmful organisms [25,26,27]. Liu et al. [28] first introduced the window concept into the grain drying field and proposed a window-control method for continuous grain drying based on equivalent accumulated temperature, marking the birth of the drying window concept. Liu et al. [29] further proposed a dual-drive mutual window AI-controlled method for continuous grain drying based on equivalent accumulated temperature and constructed a corresponding control system, which improved the control accuracy and stability of the drying process, with the outlet moisture-control accuracy ranging from −0.58% to 0.3%. Wu et al. [30] and Liu et al. [31] proposed different accurate calculation methods for actual accumulated temperature during continuous grain drying, providing technical support for the practical application of accumulated temperature in drying process control. Liu et al. [32] introduced a window-control method for continuous grain drying based on water potential accumulation and applied it to actual drying processes, which effectively improved the control accuracy of grain moisture and the quality of dried grain.

Despite the above research progress, the dynamic characteristics of drying accumulated temperature windows have not been systematically investigated, and the correlation between window states and drying process stability remains unclear. To address these gaps, this study systematically explores the variation characteristics of the equivalent window, actual window, and good window during continuous grain drying. Different from previous studies, this paper adopts matrix analysis to realize the accurate calculation of equivalent and actual accumulated temperature, proposes a quantitative judgment method for the good window, and verifies the proposed methods through simulation and physical experiments. The study takes the good window (comprehensively characterized by outlet moisture content and accumulated temperature deviation) as the core evaluation index, avoiding the one-sidedness of a single moisture content index, and connects the theoretical research of window characteristics with the practical drying. The research results demonstrate the practical application potential of accumulated temperature in grain dryers, enabling the drying process to be monitored and controlled by quantitative indicators instead of empirical or time-based judgments. The accurate calculation of equivalent and actual accumulated temperature and the establishment of the good window judgment criterion make the operation of grain dryers more stable, providing a new technical approach for the intelligent upgrading of the grain drying industry.

## 2. Materials and Methods

### 2.1. Small-Scale Continuous Drying Test System

**Figure 1:**
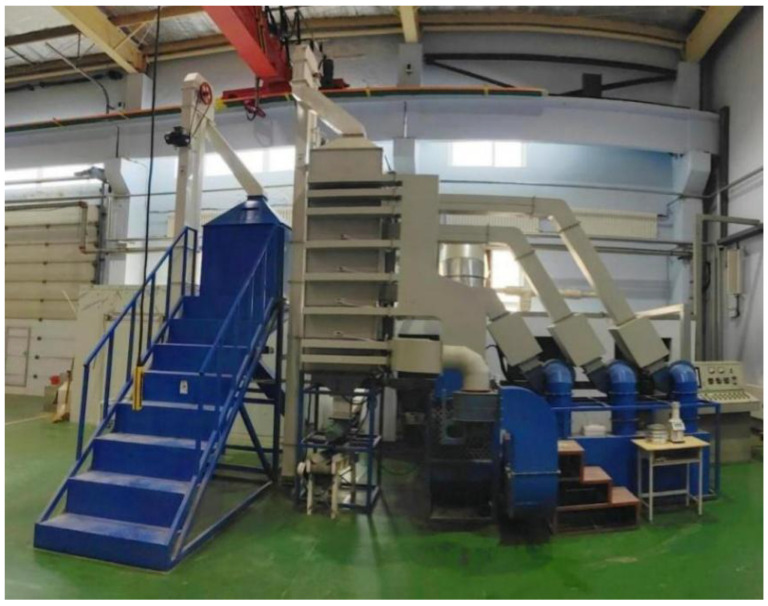
Small-scale continuous grain dryer.

**Figure 2:**
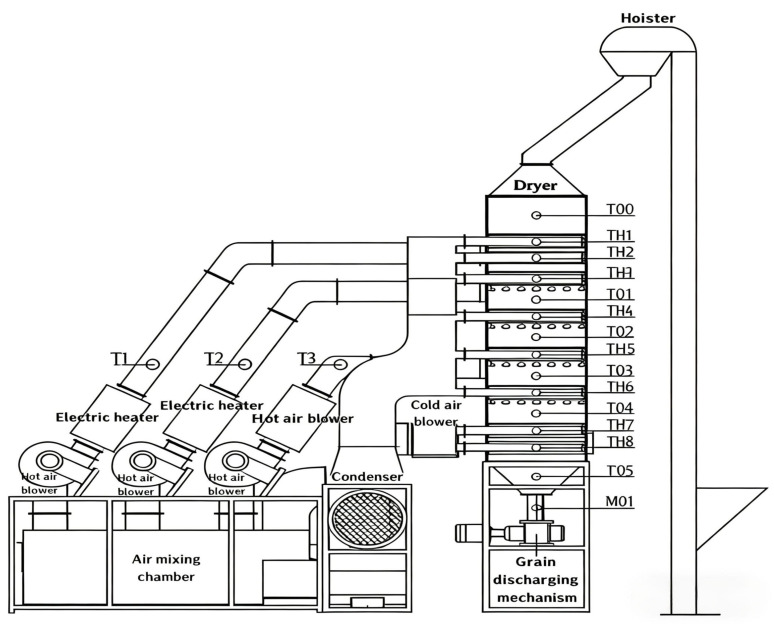
Small-scale continuous grain dryer structure and sensor layout. The sensor layout is shown in Figure 2, with hot-air temperature sensors (T1–T3), grain temperature sensors (T00–T05), tail gas temperature and humidity sensors (TH1–TH8), and a grain moisture sensor (M01).

A small-scale continuous grain dryer developed by JLU Science Instrument Equipment Co., Ltd. (Changchun, China), was used in this study. The equipment is shown in Figure 1 and mainly consists of a drying tower, heating system, transmission system, and monitoring and control system. The dryer adopts a forward and reverse-flow hot-air drying process. It is divided into six sections: grain storage section, upper drying and tempering section, middle drying and tempering section, lower drying and tempering section, cooling section, and grain discharge section. The sensor layout is shown in Figure 2, with hot-air temperature sensors (T1–T3), grain temperature sensors (T00–T05), tail gas temperature and humidity sensors (TH1–TH8), and a grain moisture sensor (M01). During grain drying, a hot-air unit delivers heated air into the drying tower to dry the grain. After passing through each drying section, grains enter the cooling section, where they are cooled, and they are then discharged from the grain discharge section, completing the drying process. To meet moisture-drying requirements, grains are discharged at regular intervals. The system can detect and automatically record, in real time, moisture content, temperature, tail gas temperature, and relative humidity at the grain outlet for automated data collection and analysis.

### 2.2. Drying Window and Drying Accumulated Temperature

In this study, the entire process of grain entering the dryer inlet to exiting the dryer outlet is defined as a drying window (*W*). For the convenience of analysis, the grain bed in the dryer is divided into several thin layers, and the hot-air state is assumed to remain constant when passing through a single thin layer and to change after completely crossing the thin layer, with the changed state acting on the next thin layer. After the drying process stabilizes, the drying process experienced by a single grain thin layer at a certain position can be regarded as the future process of the thin layer above it at a specific moment. Based on this, two core drying window types are defined: the equivalent window (*W_e_*) refers to the heating processes experienced by grain thin layers at different positions in the dryer at the same time, and the actual window (*W_a_*) refers to the continuous heating process experienced by a single grain thin layer from the top to the bottom of the dryer.

There exists a desorption equilibrium temperature in the grain-drying process. Under specific environmental conditions, grain drying and moisture reduction will stop when the heating temperature is lower than this equilibrium temperature, and the drying process will only proceed when the temperature exceeds this threshold. The desorption equilibrium temperature in drying is defined as the temperature at which grain particles reach a desorption equilibrium state with the surrounding drying medium (hot air), where the water vapor pressure on the grain surface is equal to the water vapor partial pressure in the hot air, and the moisture desorption and adsorption rates of grain reach a dynamic balance [33]. Taking the desorption equilibrium temperature of grain at a certain moment as the starting point of temperature accumulation, the total temperature that grain thin layers receive above this starting point during the drying process is defined as effective accumulated temperature (*AT*).

Each drying window in the continuous grain-drying process corresponds to a specific effective accumulated temperature, and the two core window types correspond to different accumulated temperature indices: the equivalent accumulated temperature (*AT_e_*) corresponds to the equivalent window, which refers to the accumulated temperature of a specific grain thin layer during drying and is comparable to the accumulated temperatures of other grain thin layers at different positions in the dryer at the same moment; the actual accumulated temperature (*AT_a_*) corresponds to the actual window, which refers to the total accumulated temperature experienced by the same grain thin layer at different times and positions during its movement from the top to the bottom of the dryer.

### 2.3. Calculation of Actual Accumulated Temperature

Taking the moment *t_k_* when grain is discharged from the dryer outlet as the endpoint of an actual window, a backward search is performed in the real-time data stream of the drying process (detailed specifications of the dryer are provided in Section 2.1). When the grain volume and position matching conditions are satisfied ($\sum_{i=1}^{n}Q_{i}\geq \sum_{j=1}^{N}V_{j}$), the corresponding moment *t*_1_ is the time when the grain entered the dryer inlet. The time period from the window start point *t*_1_ to the endpoint *t_k_* corresponds to the actual window (*W_a_*) of the grain batch.

The grain discharge volume during the *i*-th sampling period is calculated by Formula (1):

(1) $$Q_{i}=\frac{f_{t}}{f_{m}}\times \lambda \times V_{0}\times \frac{\tau -\tau_{w}}{\tau }$$

(2) $$\tau_{w}=\frac{AT_{0}}{\sum_{j=1}^{N}T_{j}\times \frac{V_{j}}{V_{0}}}$$

The moment when grain enters each drying section of the dryer is determined according to the following conditions:

(3) $$\left\{\begin{array}{c} \sum_{i=1}^{n_{1}}Q_{i}\geq \sum_{j=1}^{1}V_{j},\;time\;is\;t_{2}\\ \sum_{i=1}^{n_{2}}Q_{i}\geq \sum_{j=1}^{2}V_{j},\;time\;is\;t_{3}\\ \vdots \\ \sum_{i=1}^{n_{\left(N-1\right)}}Q_{i}\geq \sum_{j=1}^{N-1}V_{j},\;time\;is\;t_{N} \end{array}\right.$$

The grain temperature at different moments within the actual window is expressed as follows:

(4) $$\vec{T_{k}}=\left[\begin{array}{cccccccc} T_{t_{1},1} & \cdots & T_{t_{2},1} & \cdots & \cdots & T_{t_{i},1} & \cdots & T_{t_{k},1}\\ T_{t_{1},2} & \cdots & T_{t_{2},2} & \cdots & \cdots & T_{t_{i},2} & \cdots & T_{t_{k},2}\\ \vdots & \cdots & \vdots & \cdots & \cdots & \vdots & \cdots & \vdots \\ T_{t_{1},\left(N-1\right)} & \cdots & T_{t_{2},\left(N-1\right)} & \cdots & \cdots & T_{t_{i},\left(N-1\right)} & \cdots & T_{t_{k},\left(N-1\right)}\\ T_{t_{1},N} & \cdots & T_{t_{2},N} & \cdots & \cdots & T_{t_{i},N} & \cdots & T_{t_{k},N} \end{array}\right]$$

The sampling period of grain temperature data at different moments is expressed as follows:

(5) $$\vec{\lambda_{k}}=\left[\begin{array}{cccccccc} \lambda_{t_{1}} & \cdots & \lambda_{t_{2}} & \cdots & \cdots & \lambda_{t_{i}} & \cdots & \lambda_{t_{k}} \end{array}\right]$$

To make up for the data gap between discrete sampling points, perform interpolation $\vec{T_{k}}$ to obtain $\vec{T_{k}^{\prime }}$:

(6) $$\vec{T_{k}^{\prime }}=\left[\begin{array}{cccccccc} T_{t_{1},1} & \cdots & T_{t_{2},1} & \cdots & \cdots & T_{t_{i},1} & \cdots & T_{t_{k},1}\\ T_{t_{1},1}+\Delta_{1,1} & \cdots & T_{t_{2},1}+\Delta_{2,1} & \cdots & \cdots & T_{t_{i},1}+\Delta_{N,1} & \cdots & T_{t_{k},1}+\Delta_{k,1}\\ \vdots & \cdots & \vdots & \vdots & \vdots & \vdots & \vdots & \vdots \\ T_{t_{1},2} & \cdots & T_{t_{2},2} & \cdots & \cdots & T_{t_{i},2} & \cdots & T_{t_{k},2}\\ T_{t_{1},2}+\Delta_{1,2} & \cdots & T_{t_{2},2}+\Delta_{2,2} & \cdots & \cdots & T_{t_{i},2}+\Delta_{N,2} & \cdots & T_{t_{k},2}+\Delta_{k,2}\\ \vdots & \cdots & \vdots & \vdots & \vdots & \vdots & \vdots & \vdots \\ \vdots & \cdots & \vdots & \vdots & \vdots & \vdots & \vdots & \vdots \\ T_{t_{1},\left(N-1\right)} & \cdots & T_{t_{2},\left(N-1\right)} & \cdots & \cdots & T_{t_{i},\left(N-1\right)} & \cdots & T_{t_{k},\left(N-1\right)}\\ T_{t_{1},\left(N-1\right)}+\Delta_{1,\left(N-1\right)} & \cdots & T_{t_{2},\left(N-1\right)}+\Delta_{2,\left(N-1\right)} & \cdots & \cdots & T_{t_{i},\left(N-1\right)}+\Delta_{N,\left(N-1\right)} & \cdots & T_{t_{k},\left(N-1\right)}+\Delta_{k,\left(N-1\right)}\\ \vdots & \cdots & \vdots & \vdots & \vdots & \vdots & \vdots & \vdots \\ T_{t_{1},N} & \cdots & T_{t_{2},N} & \cdots & \cdots & T_{t_{i},N} & \cdots & T_{t_{k},N}\\ T_{t_{1},N}+\Delta_{1,N} & \cdots & T_{t_{2},N}+\Delta_{2,N} & \cdots & \cdots & T_{t_{i},N}+\Delta_{N,N} & \cdots & T_{t_{k},N}+\Delta_{k,N}\\ \vdots & \cdots & \vdots & \vdots & \vdots & \vdots & \vdots & \vdots \\ T_{t_{1},N} & \cdots & T_{t_{2},N} & \cdots & \cdots & T_{t_{i},N} & \cdots & T_{t_{k},N} \end{array}\right]$$

where Δ*_i,j_* represents the interpolated incremental data, which is calculated by Formula (7):

(7) $$\Delta_{i,j}=\frac{T_{t_{i},\left(j+1\right)}-T_{t_{i},j}}{n_{i}-n_{i-1}-1}\left(i=1,2,\cdots ,k;j=1,2,\cdots ,N\right)$$

Based on the above Formulas, the actual accumulated temperature (*AT_a_*) of the actual window is expressed as follows:

(8) $$AT_{a}=\vec{T_{dk}}\cdot \vec{\lambda_{k}^{T}}=\left[\begin{array}{cccccccc} T_{t_{1},1} & \cdots & T_{t_{2},2} & \cdots & \cdots & T_{t_{i},N} & \cdots & T_{t_{k},N} \end{array}\right]\cdot \left[\begin{array}{c} \lambda_{t_{1}}\\ \vdots \\ \lambda_{t_{2}}\\ \vdots \\ \vdots \\ \lambda_{t_{i}}\\ \vdots \\ \lambda_{t_{k}} \end{array}\right]$$

where $\vec{T_{dk}}$ represents the diagonal matrix formed by the diagonal elements of the matrix $\vec{T_{k}^{\prime }}$.

The symbols and meanings of Formulas (1)–(8) are listed in Table 1.

### 2.4. Calculation of Equivalent Accumulated Temperature

After determining the time range of a drying process window (from *t*_1_ to *t_k_*) according to Section 2.3, the equivalent time matrix for grain passing through each drying section is expressed as follows:

(9) $$\vec{\xi_{k}}=\left[\begin{array}{cccccccc} \xi_{t_{1},1} & \cdots & \xi_{t_{2},1} & \cdots & \cdots & \xi_{t_{i},1} & \cdots & \xi_{t_{k},1}\\ \xi_{t_{1},2} & \cdots & \xi_{t_{2},2} & \cdots & \cdots & \xi_{t_{i},2} & \cdots & \xi_{t_{k},2}\\ \vdots & \cdots & \vdots & \cdots & \cdots & \vdots & \cdots & \vdots \\ \xi_{t_{1},\left(N-1\right)} & \cdots & \xi_{t_{2},\left(N-1\right)} & \cdots & \cdots & \xi_{t_{i},\left(N-1\right)} & \cdots & \xi_{t_{k},\left(N-1\right)}\\ \xi_{t_{1},N} & \cdots & \xi_{t_{2},N} & \cdots & \cdots & \xi_{t_{i},N} & \cdots & \xi_{t_{k},N} \end{array}\right]$$

where $\xi_{t_{k},N}$ represents the time taken for the grain at moment *t_k_* to pass through the *N*-th drying section of the dryer, which is calculated by Formula (10):

(10) $$\xi_{t_{i},N}=\frac{V_{N}}{Q_{i}}\cdot \tau_{i}$$

The matrix $\vec{AT_{e}}$ expresses the equivalent accumulated temperature of the equivalent window, which is expressed as follows:

(11) $$\begin{array}{cc} \vec{AT_{e}}=\vec{T_{k}}\cdot \vec{\xi_{k}^{T}} & =\left[\begin{array}{cccccccc} T_{t_{1},1} & \cdots & T_{t_{2},1} & \cdots & \cdots & T_{t_{i},1} & \cdots & T_{t_{k},1}\\ T_{t_{1},2} & \cdots & T_{t_{2},2} & \cdots & \cdots & T_{t_{i},2} & \cdots & T_{t_{k},2}\\ \vdots & \cdots & \vdots & \cdots & \cdots & \vdots & \cdots & \vdots \\ T_{t_{1},\left(N-1\right)} & \cdots & T_{t_{2},\left(N-1\right)} & \cdots & \cdots & T_{t_{i},\left(N-1\right)} & \cdots & T_{t_{k},\left(N-1\right)}\\ T_{t_{1},N} & \cdots & T_{t_{2},N} & \cdots & \cdots & T_{t_{i},N} & \cdots & T_{t_{k},N} \end{array}\right]\cdot \left[\begin{array}{ccccc} \xi_{t_{1},1} & \xi_{t_{1},2} & \cdots & \xi_{t_{1},\left(N-1\right)} & \xi_{t_{1},N}\\ \vdots & \vdots & \cdots & \vdots & \vdots \\ \xi_{t_{2},1} & \xi_{t_{2},2} & \cdots & \xi_{t_{2},\left(N-1\right)} & \xi_{t_{2},N}\\ \vdots & \vdots & \cdots & \vdots & \vdots \\ \vdots & \vdots & \cdots & \vdots & \vdots \\ \xi_{t_{i},1} & \xi_{t_{i},2} & \cdots & \xi_{t_{i},\left(N-1\right)} & \xi_{t_{i},N}\\ \vdots & \vdots & \cdots & \vdots & \vdots \\ \xi_{t_{k},1} & \xi_{t_{k},2} & \cdots & \xi_{t_{k},\left(N-1\right)} & \xi_{t_{k},N} \end{array}\right]\\ & =\left[\begin{array}{ccccc} \sum_{i=1}^{k}T_{t_{i},1}\cdot \xi_{t_{i},1} & \sum_{i=1}^{k}T_{t_{i},1}\cdot \xi_{t_{i},2} & \cdots & \sum_{i=1}^{k}T_{t_{i},1}\cdot \xi_{t_{i},\left(N-1\right)} & \sum_{i=1}^{k}T_{t_{i},1}\cdot \xi_{t_{i},N}\\ \sum_{i=1}^{k}T_{t_{i},2}\cdot \xi_{t_{i},1} & \sum_{i=1}^{k}T_{t_{i},2}\cdot \xi_{t_{i},2} & \cdots & \sum_{i=1}^{k}T_{t_{i},2}\cdot \xi_{t_{i},\left(N-1\right)} & \sum_{i=1}^{k}T_{t_{i},2}\cdot \xi_{t_{i},N}\\ \vdots & \vdots & \cdots & \vdots & \vdots \\ \sum_{i=1}^{k}T_{t_{i},\left(N-1\right)}\cdot \xi_{t_{i},1} & \sum_{i=1}^{k}T_{t_{i},\left(N-1\right)}\cdot \xi_{t_{i},2} & \cdots & \sum_{i=1}^{k}T_{t_{i},\left(N-1\right)}\cdot \xi_{t_{i},\left(N-1\right)} & \sum_{i=1}^{k}T_{t_{i},\left(N-1\right)}\cdot \xi_{t_{i},N}\\ \sum_{i=1}^{k}T_{t_{i},N}\cdot \xi_{t_{i},1} & \sum_{i=1}^{k}T_{t_{i},N}\cdot \xi_{t_{i},2} & \cdots & \sum_{i=1}^{k}T_{t_{i},N}\cdot \xi_{t_{i},\left(N-1\right)} & \sum_{i=1}^{k}T_{t_{i},N}\cdot \xi_{t_{i},N} \end{array}\right] \end{array}$$

In Formula (11), the diagonal elements of the matrix are the equivalent accumulated temperature values of the grain at different moments, and the non-diagonal elements have no practical physical meaning in the drying process.

### 2.5. Judgment Criterion for the Good Window

To quantify the stable and optimal operation state of the continuous grain-drying process and provide clear and operable judgment criteria for practical drying process control and parameter optimization, this study proposes the concept of the good window (*W_g_*) and establishes its quantitative judgment method. The good window is defined as the optimal operation state window of the continuous grain-drying process, which takes the target outlet moisture content of grain and the stable deviation of accumulated temperature as the core evaluation indicators.

The specific judgment criterion for the good window is proposed as follows: the drying process is considered to enter the good window state when the dryer outlet moisture content stably maintains at 14.5 ± 0.5% (w.b.) [34], and the absolute difference between equivalent accumulated temperature and actual accumulated temperature satisfies |*AT_e_* − *AT_a_*| ≤ *δ* for more than 3 consecutive hours. Considering the inevitable measurement errors of the moisture detection sensor in practical applications, a tolerance range is set up to 20% of the continuous measurement results that are allowed to fall within the range of [13.5%, 14%] or [15%, 15.5%] (w.b.).

The establishment of this judgment method realizes the objective quantification of drying process stability, takes the maintenance of the good window state as the core goal of drying process regulation, and further connects the theoretical research of drying window characteristics (equivalent window and actual window) with the engineering application of grain drying, providing a scientific decision-making basis for the intelligent control and parameter optimization of the continuous grain-drying process.

### 2.6. Simulation of the Continuous Drying Process

#### 2.6.1. Establishment of the Continuous Corn-Drying Simulation Model

Corn is selected as the research object, and a continuous grain drying simulation model is established based on the heat and mass transfer theory of thin-layer drying to predict the drying process and key index changes under known drying conditions. The basic assumptions of the model are as follows: the thickness of the corn thin layer is ∆*x*, the time step of numerical calculation is ∆*t*, and the flow velocity of the corn deep bed in the dryer is *v_s_* (the distance that the corn thin layer moves in the time step ∆*t* is ∆*x*), which is calculated by Formula (12):

(12) $$v_{s}=\frac{\Delta x}{\Delta t}=\frac{3.2}{0.5+\tau_{w}}$$

The moisture ratio of each corn thin layer in the drying process is calculated as follows [18]:

(13) $$MR\left(j\right)=\frac{M\left(j\right)-M_{e}\left(j\right)}{M\left(j-1\right)-M_{e}\left(j\right)}=a(j)+b(j)\times \exp\left\{-{\left[K(j)\times \frac{\Delta t}{60}\right]}^{N(j)}\right\}$$

The dry basis moisture content of the corn thin layer is calculated as follows [18]:

(14) $$M\left(j\right)=\left\{a(j)+b(j)\times \exp\left\{-{\left[K(j)\times \frac{\Delta t}{60}\right]}^{N(j)}\right\}\right\}\times \left[M\left(j-1\right)-M_{e}\left(j\right)\right]+M_{e}\left(j\right)$$

The mass change in the corn thin layer caused by moisture evaporation is calculated as follows [18]:

(15) $$\Delta m(j)=\rho_{g}\times S\times \Delta x\times \frac{1}{100+M(0)}\times \left[M(j)-M(j-1)\right]$$

The moisture content of hot air after passing through each corn thin layer is calculated as follows [18]:

(16) $$H(j)=-\frac{1+H(0)}{\rho_{ma}\times S\times \Delta t\times v_{r}}\times \Delta m(j)+H(j-1)$$

The corn temperature after hot air passes through each thin layer is calculated as follows [18]:

(17) $$R(j,k)=\frac{(Q-Q_{1}-Q_{2})\times \left[100+M(0)\right]}{\rho_{g}\times S\times \Delta x\times \left[100+M(j)\right]\times C_{g}(j)}+R(j-1)$$

(18) $$Q=h\times \left[T\left(j-1\right)-R\left(j-1\right)\right]\times A\times S\times \Delta x\times \Delta t$$

(19) $$Q_{1}=-\Delta m\left(j\right)\times h_{fg}\left(j-1\right)$$

(20) $$Q_{2}=-\Delta m\left(j\right)\times C_{v}\times \left[T\left(j\right)-R\left(j-1\right)\right]$$

The temperature of hot air after passing through each thin layer is calculated as follows [18]:

(21) $$T(j)=T(j-1)-\frac{[1+H(0)]\times h\times [T(j-1)-R(j-1)]\times A\times \Delta x}{\rho_{ma}\times v_{r}\times [1+H(j-1)]\times C_{ma}(j-1)}$$

The above formulas are solved by iterative calculation until the corn thin layer at the top of the deep bed moves to the bottom of the deep bed (i.e., completes the entire drying process from inlet to outlet), and the iteration is terminated. The outlet moisture, equivalent accumulated temperature (*AT_e_*), and actual accumulated temperature (*AT_a_*) of corn after drying can be predicted by this method and the calculation methods in Section 2.3 and Section 2.4

The symbols and meanings of Formulas (12)–(21) are listed in Table 2.

#### 2.6.2. Initial Parameter Settings of Simulation for Continuous Corn-Drying Process

The variation characteristics of outlet moisture content, equivalent accumulated temperature (*AT_e_*), and actual accumulated temperature (*AT_a_*) during the drying process are simulated using MATLAB 2022. The initial parameters of the simulation model are set as follows: the cross-sectional area of the corn deep bed *S* = 0.35 m^2^, the specific surface area of corn per unit volume *A* = 784 m^2^/m^3^, the thickness of the corn thin layer ∆*x* = 20 mm, the convective heat transfer coefficient on the corn surface *h* = 56.7 W·m^2^·K^−1^, the hot-air velocity *v_r_* = 0.5 m/s, the initial corn temperature *R*(0) = 20 °C, and the initial hot-air moisture content *H*(0) = 0.01 Kg/Kg. Since the model is derived based on the thin-layer drying theory, the corn temperature is approximately equal to the hot-air temperature after the drying process stabilizes, which is quite different from the actual drying process. To reduce the simulation error, the hot-air temperature in the simulation model is set as the temperature of the dryer’s moisture exhaust port instead of the theoretical heating temperature of the hot-air unit.

#### 2.6.3. Simulation Analysis for Continuous Corn-Drying Process

**Figure 3:**
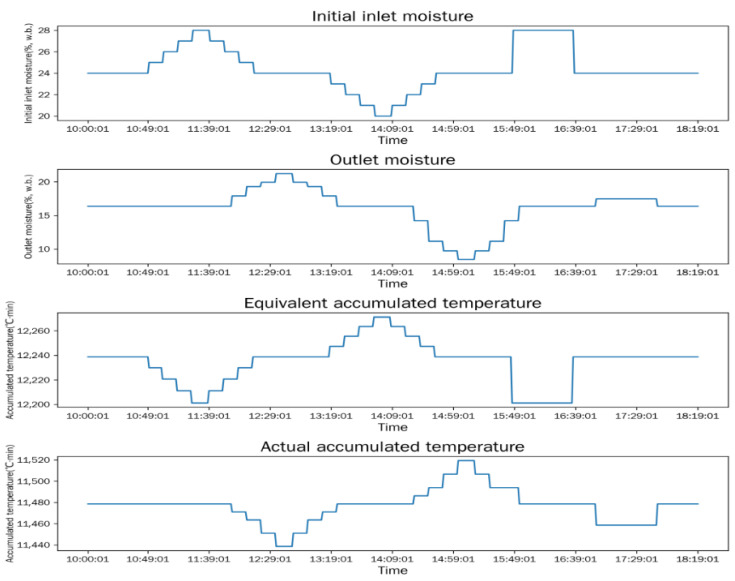
Response curves under different initial inlet moisture contents.

**Figure 4:**
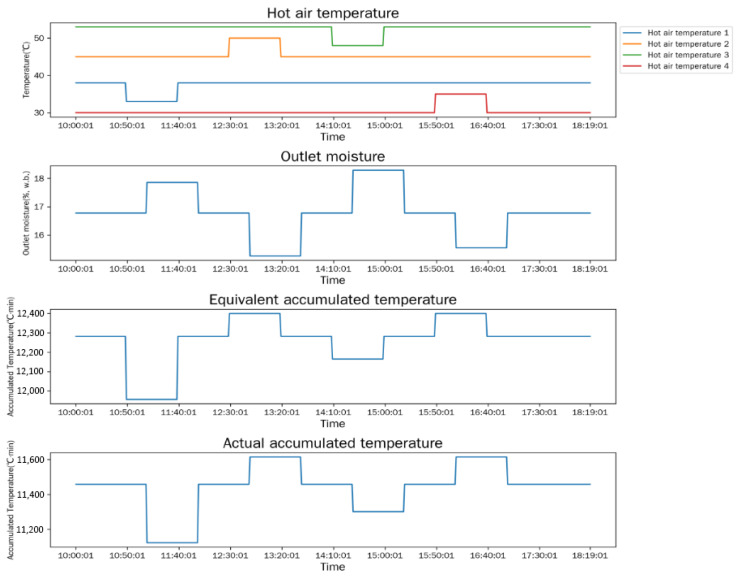
Response curves under different hot-air temperatures.

The simulation model is used to analyze the dynamic responses of outlet moisture content, equivalent accumulated temperature (*AT_e_*), and actual accumulated temperature (*AT_a_*) under two typical working condition change scenarios: different initial inlet moisture contents and different hot-air temperatures. The simulation results are shown in Figure 3 and Figure 4.

As shown in Figure 3, the equivalent accumulated temperature (*AT_e_*) changes synchronously with the variation in initial inlet moisture content, while the outlet moisture content and actual accumulated temperature (*AT_a_*) exhibit an obvious lagged response. This phenomenon is due to the real-time calculation of *AT_e_* based on the current inlet moisture content and hot-air temperature, while the outlet moisture content and *AT_a_* can only be observed and calculated after the grain batch completes the entire drying window (from inlet to outlet). *AT_e_* and *AT_a_* both show a trend of initial decrease followed by recovery: the higher the initial moisture content of corn, the more heat is required for moisture evaporation, and the heat from hot air is prioritized for evaporation, resulting in a lower corn heating rate and thus a lower accumulated temperature; with the decrease in corn moisture content, the heat used for evaporation decreases, and the corn heating rate increases, leading to the recovery of accumulated temperature [35].

As shown in Figure 4, the hot-air temperatures of the four drying sections are adjusted in sequence, and the indicators show distinct dynamic responses: when the hot-air temperature of the first drying section drops from 38 °C to 33 °C, *AT_e_* decreases immediately, *AT_a_* decreases with a time lag, and the outlet moisture content increases; when the hot-air temperature of the first section recovers to 38 °C and the second section rises from 45 °C to 50 °C, *AT_e_* increases instantaneously, the outlet moisture content decreases with a lag, and *AT_a_* also increases with a lag; the response characteristics of indicators when adjusting the hot-air temperatures of the third and fourth sections are consistent with the above rules. The simulation results further verify the instantaneous response characteristic of *AT_e_* and the lagged response characteristic of *AT_a_* to the change in drying conditions.

### 2.7. Test Conditions

#### 2.7.1. Test Parameters

The test object was corn produced in Jiutai District, Changchun City, Jilin Province, China. The drying tests are conducted in January 2023, February 2023, and March 2024, respectively, and the test parameters are shown in Table 3.

The initial moisture content of corn was measured by the oven drying method at 105 °C to constant weight [36], with three parallel measurements for each sample to ensure the accuracy of the measurement results. The tempering ratio is defined as the ratio of tempering time to drying time.

In the test, the grain discharge motor adopts an intermittent working mode: 30 s of discharge per cycle, with a real-time operating frequency of 10 Hz and a maximum frequency of 50 Hz, and the actual grain discharge volume per unit time is 32 L/min.

The specific drying process was as follows:

(1) The corn was loaded into the dryer, and the elevator was started to lift corn from the preparation warehouse to the top of the dryer. The corn entered the drying tower through the top feed inlet. Loading was stopped when the drying tower was filled.
(2) The above parameters were input into the system, the dryer was then started, and the control system automatically collected sensor data and performed real-time online control (drying conditions and test data were collected at 1 min intervals).
(3) After all the dried corn was discharged from the dryer, the test was completed, and the equipment was shut down.

**Figure 5:**
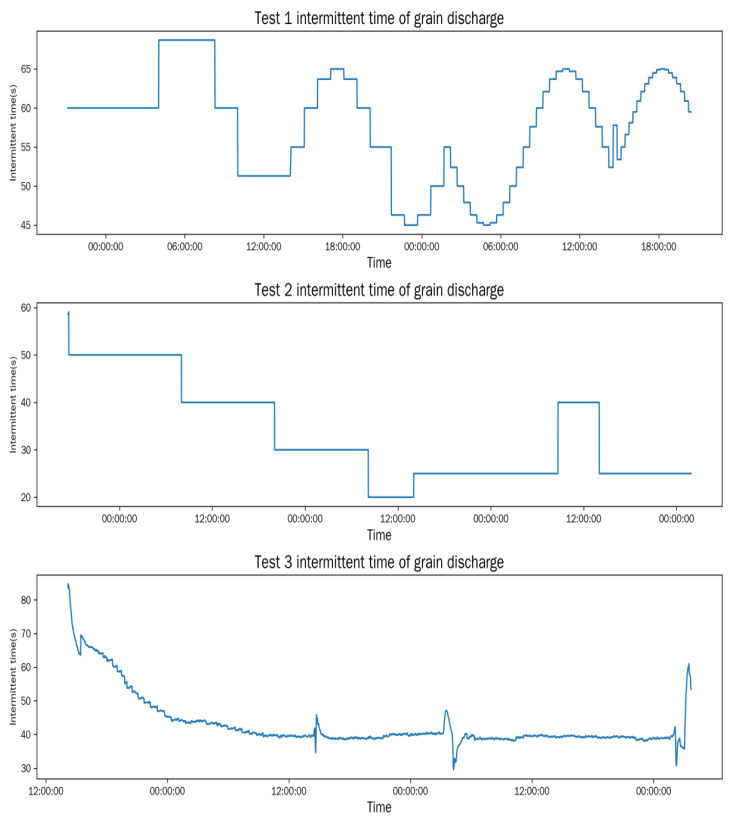
Intermittent grain discharge time.

The grain discharge intervals for each test are shown in Figure 5.

#### 2.7.2. Test Indicators and Evaluation Methods

Based on the simulation results in Figure 3 and Figure 4, the difference between the simulated values of equivalent accumulated temperature and actual accumulated temperature is approximately 1000 °C·min. Considering the measurement errors and working condition fluctuations in the physical experiment, the optimal judgment threshold *δ* of the accumulated temperature difference is adjusted to 1500 °C·min. The final good window judgment criterion applied in the physical test is as follows: the dryer outlet moisture content stably maintains at 14.5 ± 0.5% (w.b.), and the absolute difference between equivalent accumulated temperature and actual accumulated temperature satisfies |*AT_e_* − *AT_a_*| ≤ 1500 °C·min for more than 3 consecutive hours.

The outlet grain moisture content (%, w.b.) was automatically monitored by the capacitive moisture online detector installed on the dryer, with a precision of ±0.5% [37]. The moisture content was the average of three measurements, rounded to two decimal places.

The dry basis moisture content of corn (%, d.b.) is calculated as follows [38]:

(22) $$M=\frac{M_{t}}{100-M_{t}}\times 100$$

In Formula (22), *M_t_* is the wet basis moisture content of corn (%, w.b.).

The mean relative error (MRE) is used to evaluate the prediction accuracy of the simulation model, which measures the relative deviation between the simulated predicted value and the actual measured value of each parameter (equivalent accumulated temperature, actual accumulated temperature, and outlet moisture content).

The calculation formula can be expressed as:

(23) $$MRE=\frac{1}{n}\sum_{i=1}^{n}\left|\frac{y_{i}-{\hat{y}}_{i}}{y_{i}}\right|\times 100\%$$

In Formula (23), *y_i_* is the actual value, ${\hat{y}}_{i}$ is the predicted value, and *n* is the sample size.

## 3. Results

### 3.1. Analysis of Simulation and Actual Drying Results of Test 1

In Test 1, the intermittent grain discharge time is controlled by a sine function to clearly and intuitively observe the dynamic variation characteristics of the actual window (*W_a_*) and equivalent window (*W_e_*) during the grain-drying process. Since they cannot be directly observed, their variations are characterized using the actual accumulated temperature (*AT_a_*) and equivalent accumulated temperature (*AT_e_*). The test focuses on the dynamic response of the windows, so the dryer outlet moisture content is not required to be within the target range, and thus, the moisture data is not recorded for quantitative analysis.

**Figure 6:**
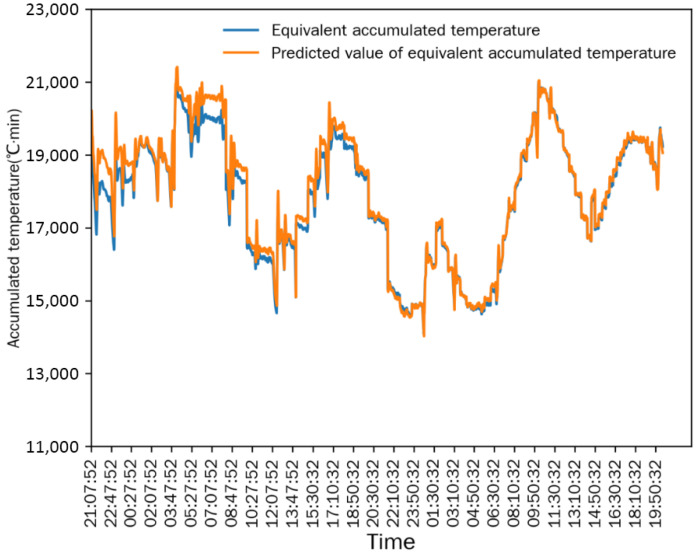
Comparison between equivalent accumulated temperature and predicted value in Test 1.

**Figure 7:**
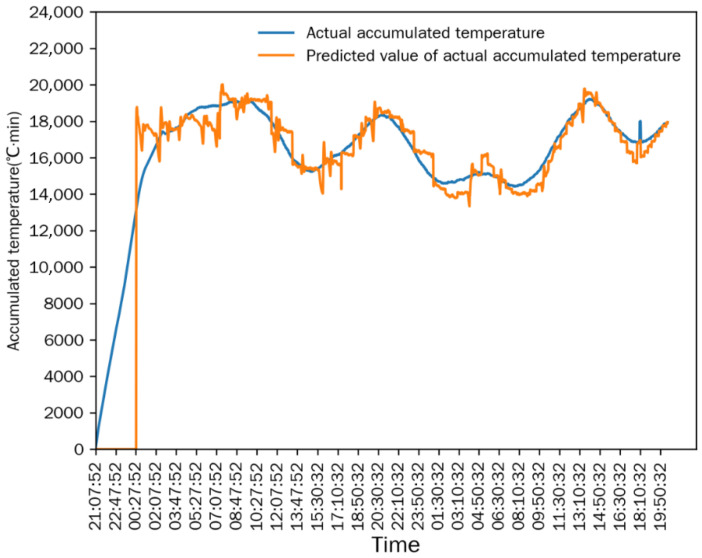
Comparison between actual accumulated temperature and predicted value in Test 1.

As shown in Figure 6 and Figure 7, after the drying process stabilized, the differences between the measured value and the simulated predicted value of the equivalent accumulated temperature (*AT_e_*) and actual accumulated temperature (*AT_a_*) were extremely small, and the change trends of the two were completely consistent. This indicated that the numerical simulation method established in Section 2.6 could accurately simulate the continuous corn-drying process, and the simulation results were highly consistent with the actual measured results. The mean relative error (MRE) is used for the statistical analysis of the simulation accuracy: the MRE between the measured value and predicted value of *AT_e_* was 1.1677%, and the MRE between the measured value and predicted value of *AT_a_* was 3.0116%. The small mean relative errors demonstrate the high prediction accuracy of the simulation model.

**Figure 8:**
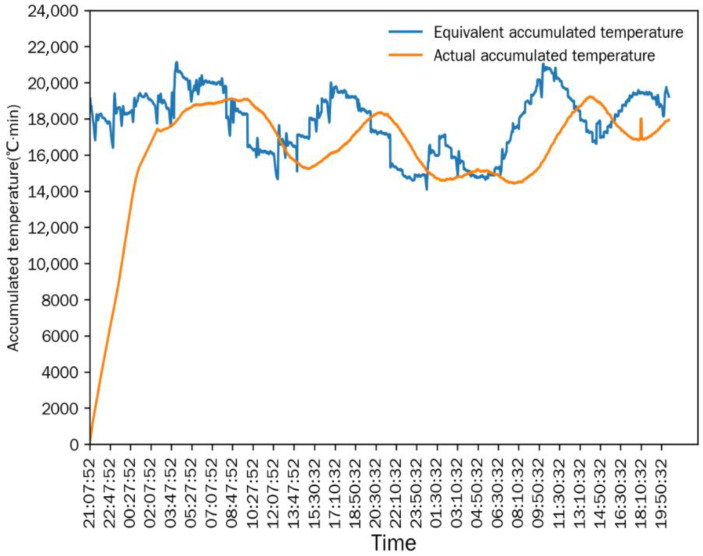
Comparison between equivalent accumulated temperature and actual accumulated temperature in Test 1.

Figure 8 shows the dynamic variation characteristics of *AT_e_* and *AT_a_* during the corn-drying process. *AT_a_* showed a gradual increasing trend with the progress of the drying process, while *AT_e_* was calculated in real-time based on the collected multi-position sensor data, showing a dynamic fluctuation trend. After the drying process stabilized, the absolute difference between *AT_e_* and *AT_a_* gradually decreased and maintained within 1500 °C·min, indicating that the corn batch entering from the dryer inlet had completed the entire drying window and been discharged from the outlet, and a complete actual window had been formed. Since the intermittent grain discharge time was controlled by a sine function, *AT_e_* and *AT_a_* also showed corresponding sine fluctuations. The key finding is that *AT_a_* has an obvious time lag relative to *AT_e_*, and the lag time is exactly equal to the time required for grain to complete one drying cycle (from inlet to outlet), i.e., one drying window.

### 3.2. Analysis of Simulation and Actual Drying Results of Test 2

In Test 2, the intermittent grain discharge time is adjusted according to specific rules, and the dynamic changes in the actual window (*W_a_*), equivalent window (*W_e_*), and good window (*W_g_*) are observed at the same time. The variations in the actual window (*W_a_*) and equivalent window (*W_e_*) are characterized by the actual accumulated temperature (*AT_a_*) and equivalent accumulated temperature (*AT_e_*). After the drying process enters the good window state and stabilizes for a certain period, the intermittent grain discharge time is artificially adjusted to destroy the good window state; then, the time required for the drying process to recover to the good window state again is observed.

**Figure 9:**
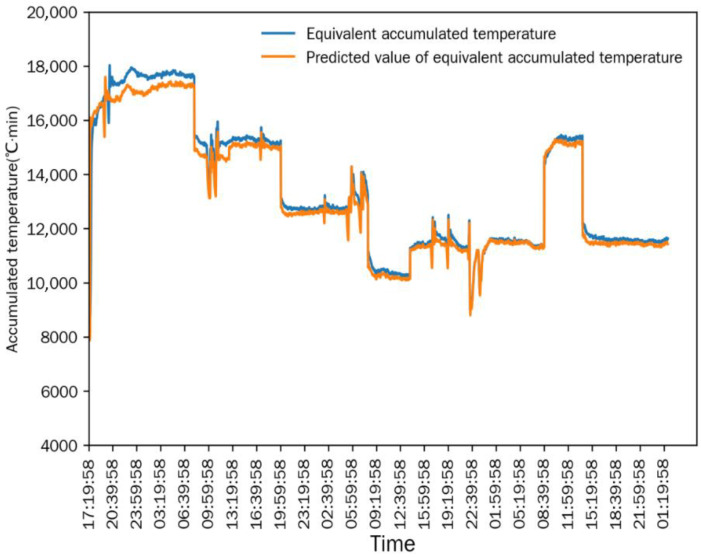
Comparison between equivalent accumulated temperature and predicted value in Test 2.

**Figure 10:**
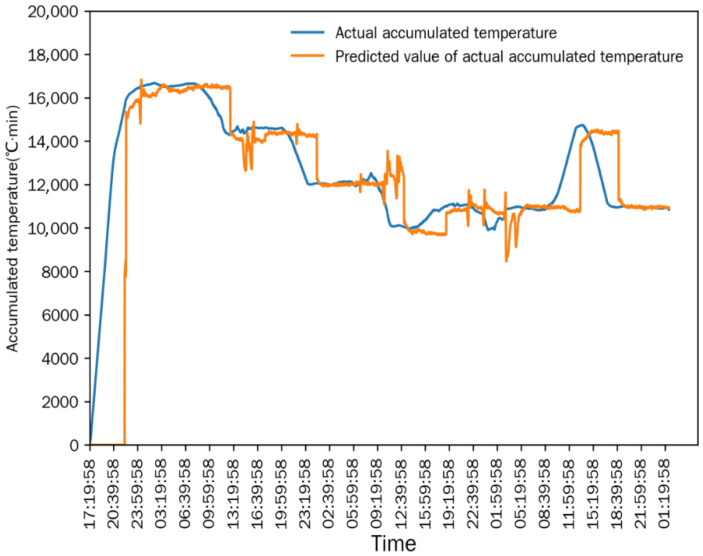
Comparison between actual accumulated temperature and predicted value in Test 2.

**Figure 11:**
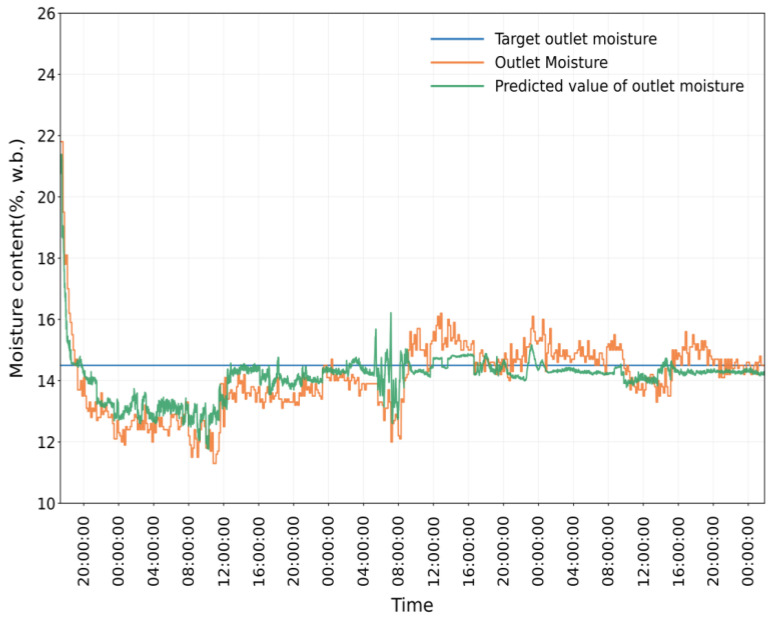
Comparison between outlet moisture and predicted value in Test 2.

As shown in Figure 9, Figure 10 and Figure 11, the simulation model still showed good prediction performance in Test 2. The MRE between the measured value and predicted value of the equivalent accumulated temperature (*AT_e_*) was 1.7842%, the MRE between the measured value and predicted value of the actual accumulated temperature (*AT_a_*) was 5.5060%, and the MRE between the measured value and predicted value of the outlet moisture was 4.1001%. At the initial stage of drying, the measured value of outlet moisture content has a large deviation from the target value, which is due to the instability of the drying process (e.g., the fluctuation of hot-air temperature and humidity, the uneven distribution of grain in the drying tower). With the gradual stabilization of the drying process, the deviation between the measured value and the predicted value of outlet moisture content was significantly reduced. The mean relative error of each sampling point was small, indicating that the simulation model had high prediction accuracy.

**Figure 12:**
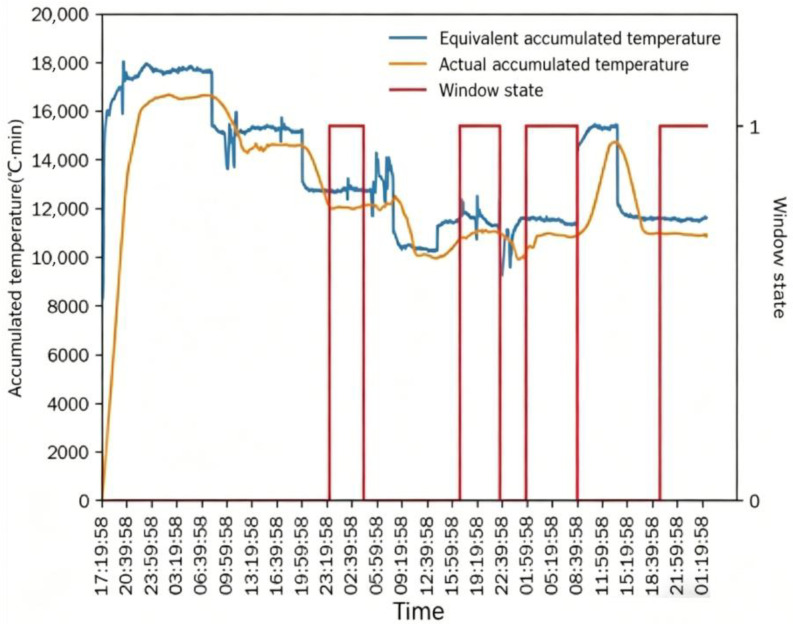
Accumulated temperature comparison and window state in Test 2.

In Figure 11 and Figure 12, *AT_e_* increased rapidly at the initial stage of drying, while *AT_a_* increased gradually with the drying process. After the drying process stabilized, the absolute difference between *AT_e_* and *AT_a_* stabilized and was controlled within 1500 °C·min, which meets the good window judgment criterion. After maintaining the good window state for a certain period, the window state changed continuously with the adjustment of intermittent grain discharge time, and the drying process entered the good window state for three consecutive periods. The dryer outlet moisture content also changed with the adjustment of intermittent grain discharge time, and the variation trend was consistent with the window state. Similar to Test 1, *AT_e_* and *AT_a_* both changed with the adjustment of intermittent grain discharge time, and the time lag of *AT_a_* relative to *AT_e_* was exactly one drying window.

The good window (*W_g_*) only appeared after the drying process completed a full drying cycle and stabilized for a certain period, which verified the rationality of the 3 h stability requirement in the good window judgment criterion. When *W_g_* was destroyed by adjusting the intermittent grain discharge time, *AT_e_* responded immediately to the change in working conditions, while *AT_a_* showed an obvious lagged response, and *W_g_* could only be recovered after the drying process re-stabilized.

### 3.3. Analysis of Simulation and Actual Drying Results of Test 3

In Test 3, the intermittent grain discharge time of the dryer is calculated and adjusted in real-time by the control system without artificial intervention, which is more consistent with the actual working conditions of commercial grain dryers. The test objective is to observe the variation characteristics of the three types of windows under the condition of automatic control and to further verify the universality of the good window judgment criterion. Similar to Section 3.1 and Section 3.2, the variations in the actual window (*W_a_*) and equivalent window (*W_e_*) are characterized by the actual accumulated temperature (*AT_a_*) and equivalent accumulated temperature (*AT_e_*). The good window (*W_g_*) is judged according to the good window judgment criterion in Section 2.5.

**Figure 13:**
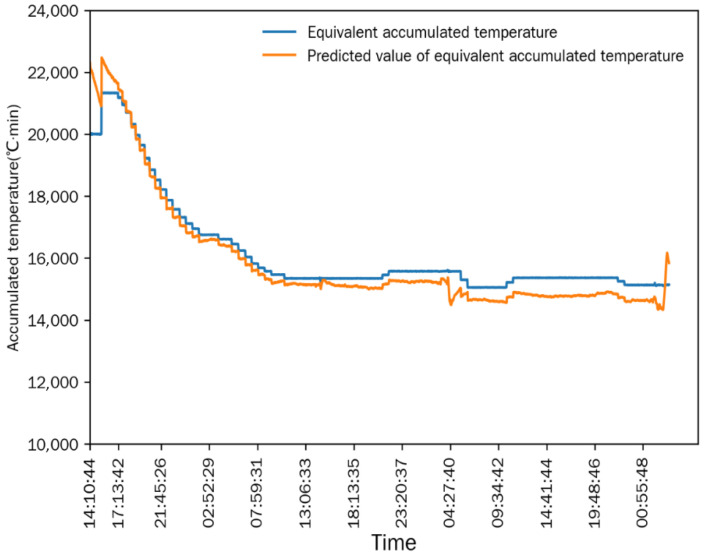
Comparison between equivalent accumulated temperature and predicted value in Test 3.

**Figure 14:**
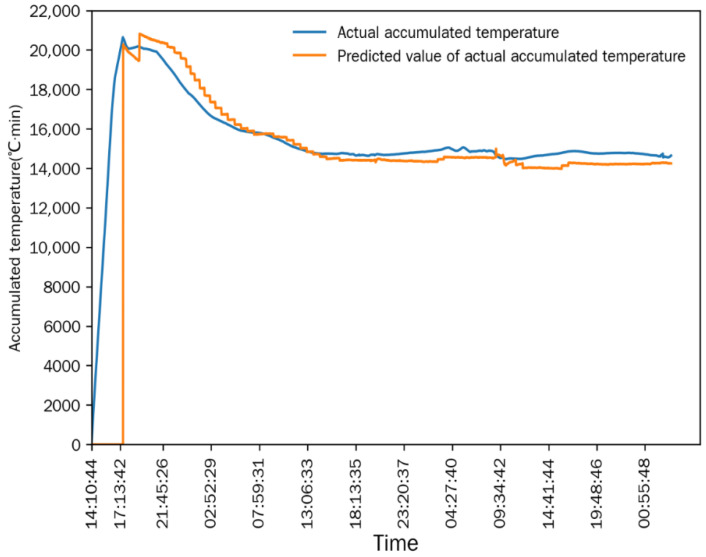
Comparison between actual accumulated temperature and predicted value in Test 3.

**Figure 15:**
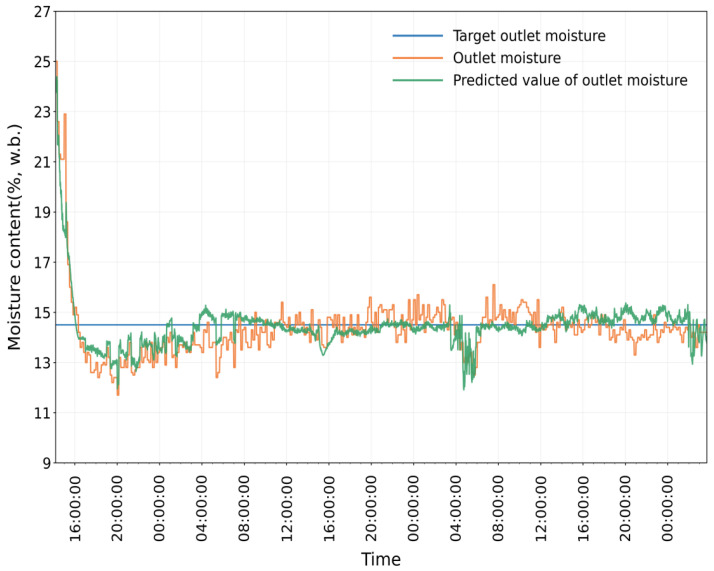
Comparison between outlet moisture and predicted value in Test 3.

As shown in Figure 13, Figure 14 and Figure 15, the simulation results in Test 3 were consistent with those in the previous two tests, and the simulated predicted values were highly consistent with the actual measured values. The MRE between the measured value and predicted value of the equivalent accumulated temperature (*AT_e_*) was 2.4985%, the MRE between the measured value and predicted value of the equivalent actual temperature (*AT_a_*) was 2.5834%, and the MRE between the measured value and predicted value of the outlet moisture was 4.5917%. The small mean relative errors indicate that the simulation model had a good fitting effect and strong universality under automatic control conditions.

**Figure 16:**
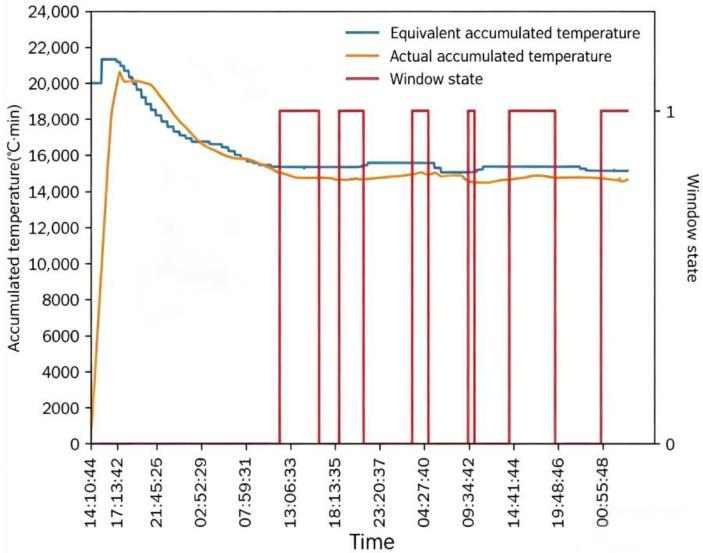
Accumulated temperature comparison and window state in Test 3.

In Figure 15 and Figure 16, after the drying process stabilized, the maximum absolute difference between *AT_e_* and *AT_a_* was 1361.7 °C·min, and most of the time was within 1000 °C·min, which was smaller than the difference in the previous two tests. This is due to the real-time adjustment of the intermittent grain discharge time by the automatic control system, which makes the drying process more stable. With the gradual decrease in the intermittent grain discharge time, *AT_e_* and *AT_a_* showed a synchronous decreasing trend, and *AT_a_* still had a time lag of one drying window relative to *AT_e_*. The outlet moisture content gradually approached the target value (14.5%) with the decrease in the intermittent grain discharge time and fluctuated stably around the target value. This phenomenon is the result of the automatic control system’s self-regulation of the drying process.

The window state changed multiple times. Theoretically, once the drying process enters the good window (*W_g_*), the subsequent window state should remain stable if the working conditions do not change. However, in the actual drying process, there are many uncontrollable interference factors, such as the fluctuation of inlet moisture content and environmental temperature/humidity, the measurement error of the moisture sensor, and the uneven flow of hot air, which lead to the fluctuation of the window state. Similar to Tests 1 and 2, *W_g_* only appeared after the drying process stabilized; when the intermittent grain discharge time changed under automatic control, *AT_e_* responded quickly to the change, *AT_a_* responded with a lag, and *W_g_* could only recover after the drying process re-stabilized.

## 4. Discussion

This study systematically explores the dynamic characteristics of the equivalent window (*W_e_*), actual window (*W_a_*), and good window (*W_g_*) during continuous grain drying, establishes the accurate calculation methods of equivalent accumulated temperature (*AT_e_*) and actual accumulated temperature (*AT_a_*) based on matrix analysis, and proposes a feasible judgment criterion for the good window. The simulation and experimental results verify the validity and practicability of the proposed methods and criteria, and provide valuable references for the optimization of the continuous grain-drying process.

The significant time lag between the actual window and the equivalent window is the core discovery of this study. This lag effect is consistent with the inherent transmission characteristics of the grain-drying process: grain moisture migration and heat transfer are gradual physical processes, and the change in inlet working conditions (e.g., moisture content, temperature) cannot immediately affect the outlet state, which results in the lag of the actual window relative to the equivalent window. The good window judgment method proposed in this study—taking outlet moisture stabilization at 14.5 ± 0.5% and |*AT_e_* − *AT_a_*| ≤ 1500 °C·min for more than 3 h as the criteria—has been effectively verified in tests. In the subsequent extended test of another type of commercial grain dryer, we found that this judgment criterion still has good applicability, and the allowable difference between equivalent accumulated temperature and actual accumulated temperature is even reduced to ± 500 °C·min due to the higher stability of the commercial dryer. This criterion comprehensively considers both the outlet moisture and the accumulated temperature deviation, avoiding the one-sidedness of judging the drying process only by the moisture content index, and has strong adaptability to the dynamic changes in working conditions in the actual drying process. The established MATLAB simulation model shows high prediction accuracy, with the mean relative errors (MREs) of equivalent accumulated temperature, actual accumulated temperature, and outlet moisture all maintained at approximately 5%. The model can accurately reproduce the dynamic variation characteristics of indicators during the continuous corn-drying process. Compared with the existing grain drying models, this study innovatively introduces the concept of drying window characteristics into the simulation model, enabling the simultaneous prediction of the dynamic changes in accumulated temperature and moisture content, and realizing the quantitative characterization of the drying process state by the window state.

Despite the positive research results, this study still has certain limitations, which also point out the direction for subsequent research. First, this study only takes corn as the drying research object, and the proposed good window judgment criterion is mainly applicable to corn drying. Subsequent research should expand the scope of test objects to include other main food grains (such as rice, wheat, and soybean), adjust the accumulated temperature threshold and moisture-control range according to the unique drying characteristics of different grains, and improve the universality of the window characteristic theory and good window judgment criterion. Second, the established simulation model is based on the thin-layer drying theory, and the heat loss of the dryer is not considered in the model. In the actual drying process, the heat loss of the dryer casing and pipeline is an important factor affecting the accumulated temperature and drying efficiency, especially for large-scale commercial dryers. Future research should refine the heat and mass transfer mechanism in the model, introduce the dynamic change in hot-air parameters and the heat loss model of the dryer, and further improve the simulation accuracy of the model. Finally, this study mainly focuses on the influence of intermittent grain discharge time and hot-air temperature on window characteristics, and other key drying parameters (such as hot-air velocity, tempering time, and grain layer thickness) are set as constant values. In the actual drying process, these parameters also have an important impact on the drying process and window characteristics, and there is a complex interaction between multiple parameters. Future research can explore the interaction mechanism between multiple drying parameters and window characteristics, establish a multi-factor optimization model for the good window design, and realize the precise control of the drying process under complex working conditions.

## 5. Conclusions

This study systematically investigated the window characteristics in the continuous grain-drying process, developed accurate calculation methods for equivalent and actual accumulated temperature based on matrix analysis, and proposed a feasible and practical good window judgment criterion. A MATLAB-based simulation model for continuous corn drying was constructed, and the validity and practicability of the proposed methods and criteria were verified through three sets of drying tests.

Three types of drying windows (equivalent window, actual window, and good window) in the continuous grain-drying process exhibited distinct dynamic response characteristics: the equivalent accumulated temperature responded instantaneously to changes in drying conditions (e.g., hot-air temperature, inlet moisture content); the actual accumulated temperature increased progressively with the drying process and had a significant time lag relative to equivalent accumulated temperature, with the lag time exactly equal to one complete drying cycle. Upon process stabilization, the absolute difference between equivalent and actual accumulated temperature was controlled within 1500 °C·min or even smaller. A drying process was identified to enter the good window state when the outlet moisture content was stably maintained at 14.5 ± 0.5% (w.b.) and the accumulated temperature difference satisfied |*AT_e_* − *AT_a_*| ≤ 1500 °C·min for more than three consecutive hours. The adjustment of intermittent grain discharge time induced dynamic responses of the drying windows: the equivalent accumulated temperature adjusted rapidly to the change in grain discharge time, while the actual accumulated temperature and the good window state showed a lagged response and could only recover to the stable state after the drying process re-stabilized. This result highlighted the necessity of real-time parameter regulation based on the dynamic characteristics of drying windows in the actual drying process, which could effectively reduce the re-stabilization time of the drying process and improve the overall drying efficiency.

The established MATLAB simulation model achieved high prediction accuracy. The mean relative errors (MRE) of equivalent accumulated temperature for each group were 1.1677%, 1.7842%, and 2.4985%, respectively; those of actual accumulated temperature were 3.0116%, 5.5060%, and 2.5834%, respectively; and those of outlet moisture were 4.1001% and 4.5917%, respectively. All MRE values were maintained at approximately 5%, and the model could accurately reproduce the dynamic variation characteristics of the drying process and window states, providing a reliable technical tool for the pre-evaluation, operational optimization, and control strategy design of the grain-drying system.

## Figures and Tables

**Table 1:** Formulas (1)–(8) symbols and meanings.

Symbol	Unit	Meaning
*Q_i_*	L	The volume of grains discharged from the dryer during the *i*-th sampling period
*V_j_*	L	The volume in the *j*-th drying section
*T_j_*	°C	The temperature of grains in the *j*-th drying section
*V*_0_	L/min	The volume of grains discharged per unit time
*f_t_*	Hz	The real-time discharge motor frequency of grains
*f_m_*	Hz	The maximum discharge motor frequency of grains
*τ*	min	The discharge cycle of grains
*τ_w_*	min	The intermittent discharge time of grains
*λ*	min	The data sampling period
*AT*_0_	°C·min	The initial theoretical accumulated temperature of grains
*t*_2_, …, *t_N_*	/	The time when grains enter the 2nd, …, *N*-th drying section
$\lambda_{t_{i}}$	min	The data sampling period at time *t_i_*
$T_{t_{i},N}$	°C	The temperature of grains at time *t_i_* in the *N*-th drying section

**Table 2:** Formulas (12)–(21) symbols and meanings.

Symbol	Unit	Meaning
∆*x*	mm	The thickness of the corn thin layer
∆*t*	min	The time step
*v_s_*	m/s	The flow velocity of the corn deep bed
*v_r_*	m/s	The hot-air velocity
*τ_w_*	min	The intermittent discharge time
*a*, *b*, *K*, *N*	/	The drying constants
*S*	m^2^	The cross-sectional area of the corn deep bed
*A*	m^2^/m^3^	The surface area per unit volume of corn
*ρ_g_*	Kg/m^3^	The density of wet corn
*ρ_ma_*	Kg/m^3^	The density of wet air
*C_g_*	J·Kg^−1^·K^−1^	The specific heat capacity of wet corn
*C_ma_*	J·Kg^−1^·K^−1^	The specific heat capacity of wet air
*C_v_*	J·Kg^−1^·K^−1^	The specific heat capacity of water vapor
*h_fg_*	J/Kg	The heat of vaporization of water
*h*	W·m^2^·K^−1^	The convective heat transfer coefficient on the corn surface
*Q*	J	The heat is transferred from the corn by hot air through convection
*Q*_1_	J	The heat for evaporating moisture in the corn thin layer
*Q*_2_	J	The heat for increasing the temperature of water vapor
*MR*(*j*)	/	The moisture ratio in the *j*-th thin layer
*M_e_*(*j*) (d.b.)	%	The equilibrium dry basis moisture content of corn in the *j*-th thin layer
*M*(*j*) (d.b.)	%	The dry basis moisture content of corn in the *j*-th thin layer
*H*(*j*)	Kg/Kg	The moisture content of hot air in the *j*-th thin layer
*R*(*j*)	°C	The temperature of corn in the *j*-th thin layer
*T*(*j*)	°C	The temperature of the hot air in the *j*-th thin layer

**Table 3:** Test parameters of the continuous corn drying.

Test Parameters	Test 1	Test 2	Test 3
Initial moisture content (%, w.b)	24.2	21.8	25.0
Tempering ratio	3:1	3:1	3:1
Hot-air temperature 1 (°C)	110	110	110
Hot-air temperature 2 (°C)	100	100	100
Hot-air temperature 3 (°C)	90	90	90
Hot-air relative humidity (%)	37.1~52.2	34.9~51.6	38.1~54.8
Environmental temperature (°C)	15.2~19.2	14.7~18.6	15.1~20
Environmental relative humidity (%)	33.5~43.5	28.4~41.0	30.2~46.8
Hot-air velocity (m/s)	0.8~1.3	0.8~1.3	0.8~1.3
Target outlet moisture content (%, w.b.)	14.5	14.5	14.5
Outlet moisture content threshold (%, w.b.)	0.5	0.5	0.5

## Data Availability

The raw data supporting the conclusions of this article will be made available by the authors on request.

## References

[1] Chen T., Huang Q., Gao D., Huang Z., Zheng Y., Li Y. (2002). Accumulated Temperature as an Indicator to Predict the Stabilizing Process in Sewage Sludge Composting. Acta Ecol. Sin..

[2] Zheng D., Sun Z. (2010). Discussion on Scientificalness Problem of Accumulated Temperature and Its Unit. Chin. J. Agrometeorol..

[3] Sacks W.J., Kucharik C.J. (2011). Crop Management and Phenology Trends in The U.S. CornBelt: Impacts on Yields, Evapotranspiration and Energy Balance. Agric. For. Meteorol..

[4] Shao R., Yu K., Li H., Jia S., Yang Q., Zhao X., Zhao Y., Liu T. (2021). The effect of elevating temperature on the growth and development of reproductive organs and yield of summer maize. J. Integr. Agric..

[5] Paredes P., Lopez-Urrea R., Martinez-Romero A., Petry M.T., Cameira M.D., Montoya F., Almeida W., Salman M., Pereira L.S. (2025). Base and upper temperature thresholds to support the calculation of growing degree days aiming at their use with the FAO56rev crop coefficients curve: A review. Agric. Water Manag..

[6] Murakami T., Doi Y., Morita H. (2009). Relation between the accumulated temperature in effective degrees and the ripening of rice. Chemosphere.

[7] Liu Y., Hou P., Xie R., Hao W., Li S., Mei X. (2015). Spatial Variation and Improving Measures of the Utilization Efficiency of Accumulated Temperature. Crop Sci..

[8] Wen H., Wu T., Jia H., Song W., Xu C., Han T., Sun S., Wu C. (2022). Analysis of Relationship between Soybean Relative Maturity Group, Crop Heat Units and ≥10 °C Active Accumulated Temperature. Agronomy.

[9] Dong J., Liu J., Tao F., Xu X., Wang J. (2009). Spatio-temporal changes in annual accumulated temperature in China and the effects on cropping systems, 1980s to 2000. Clim. Res..

[10] Paredes P., López-Urrea R., Martinez-Romero A., Petry M., Cameira M.D., Montoya F., Salman M., Pereira L.S. (2025). Estimating the lengths of crop growth stages to define the crop coefficient curves using growing degree days (GDD): Application of the revised FAO56 guidelines. Agric. Water Manag..

[11] Ni X., Gunawan G., Brown S.L., Sumner P.E., Ruberson J.R., Buntin G.D., Holbrook C.C., Lee R.D., Streett D.A., Throne J.E. (2008). Insect-Attracting and Antimicrobial Properties of Antifreeze for Monitoring Insect Pests and Natural Enemies in Stored Corn. J. Econ. Entomol..

[12] Li D., Kang Z., Wang J., Wang H., Dong J., Liang S. (2010). Threshold temperature and effective accumulated temperature of peach fruit borer, *Carposina sasakii*. Chin. Bull. Entomol..

[13] Wu W., Jin Y., Qi D., Wang X., Zhang Y., Han F., Xu Y., Wang R. (2018). A Measurement and Control Method for Continuous Grain Drying Based on Equivalent Accumulated Temperature. Chinese Patent.

[14] Wu W., Cui H., Jin Y., Liu Z., Han F., Xu Y., Zhang Y., Cheng Z. (2018). A Measurement and Control Method for Batch-Type Static Bed Grain Drying Process Based on Equivalent Accumulated Temperature. Chinese Patent.

[15] Liu Z., Wu Z., Wu W., Jin Y., Wu Y., Han F., Zhang Y., Xu Y., Cui H., Xing Z. (2018). A Method for Measuring Equivalent Accumulated Temperature During the Continuous Grain Drying Process. Chinese Patent.

[16] Liu Z., Wu Z., Wu W., Jin Y., Han F., Zhang Y., Xu Y., Cui H. (2018). A Measurement and Control Method and Device for Grain Circulation Drying Based on Equivalent Accumulated Temperature. Chinese Patent.

[17] Wu Y. (2017). Study on Temperature-Accumulation Quality Properties of Grain with Measurement and Control Method in Drying Process. Ph.D. Thesis.

[18] Qi D. (2018). Establishment and Application of Water Prediction and Control Model for Continuous Corn Dryer. Master’s Thesis.

[19] Wang X. (2018). Study on the Measurement and Control System of Continuous Maize Dryers. Master’s Thesis.

[20] Jin Y. (2019). Modeling and Intelligent Control of Grain Drying Process Based on Equivalent Accumulated Temperature. Ph.D. Thesis.

[21] Wu Y., Fu D., Yin H., Xu X., Zhao C., Liu J., Wu W. (2020). Establishment of Mathematical Model of Accumulated Temperature of Corn Based on Multi-Parameter Controllable Thin-Layer Drying Experiment and Its Tool Chart. J. Chin. Cereals Oils Assoc..

[22] Li J., Yin J., Jin Y., Yi X., Zhang Z. (2024). Study on Optimization of Hot Air Drying Process and Nutrient Quality Model of Rice Driven by Accumulated Temperature. Sci. Technol. Cereals Oils Foods.

[23] Jin Y., Wong K., Wu Z., Qi D., Wang R., Han F., Wu W. (2019). Relationship between accumulated temperature and quality of paddy. Int. J. Food Prop..

[24] Jin Y., Yin J., Xie H., Zhang Z. (2021). Reconstruction of rice drying model and analysis of tempering characteristics based on drying accumulated temperature. Appl. Sci..

[25] Li X., Wu X., Yin S. (2014). Lowering paddy temperature by equilibrium moisture theory and ventilation window. Sci. Technol. Cereals Oils Foods.

[26] Li X., Wu Z., Ji Z., Yang X., Zhao Y., Yan E., Wu X. (2017). Equilibrium Moisture Content (EMC) of Chinese Wheat and Lowering Grain Temperature with Mechanical Aeration Guided by EMC Theory. J. Chin. Cereals Oils Assoc..

[27] Chen L., Wu W., Qin X., Wu Y., Chen S., Liu J., Zhang Y. (2015). Remote intelligent measurement and control system for granary ventilation based on absolute water potential diagram. Trans. Chin. Soc. Agric. Eng..

[28] Liu Z., Wu W., Han F., Xu Y., Jin Y., Wu Z. (2019). A Window Control Method for Continuous Grain Drying Based on Equivalent Accumulated Temperature. Chinese Patent.

[29] Liu Z., Xu Y., Han F., Zhang Y., Wang G., Wu Z., Wu W. (2022). Control Method for Continuous Grain Drying Based on Equivalent Accumulated Temperature Mechanism and Artificial Intelligence. Foods.

[30] Wu W., Song J., Jin X., Liu Z., Han F., Zhang J., Wu Y., Yu Z., Zhang Y., Wang G. (2024). A Method for Calculating Actual Accumulated Temperature Suitable for Process Control of Continuous Grain Dryer. Chinese Patent.

[31] Liu Z., Jin X., Wang G., Wu W., Zhang J., Wu Y., Yu Z., Zhang Y., Han F., Song J. (2024). A Method for Accurate Calculation of Actual Accumulated Temperature Suitable for Process Control of a Continuous Grain Dryer. Chinese Patent.

[32] Liu Z., Jin X., Chen J., Wu W., Han F., Xu Y. (2025). Dual-Drive Window Control Method for Continuous Grain Drying Based on Water Potential Accumulation. Agriculture.

[33] Chen S., Wu W., Li X., Wu Z., Zhang Y., Zhang Z., Han F. (2016). A new model of grain moisture heat balance and its thermodynamic properties. J. Chin. Cereals Oils Assoc..

[34] Liu S., Zhao Y., Liu X., Gong Z., Lv H. (2024). Effects of Different Temperatures on the Storage Quality of Maize with High Moisture Content. Food Res. Dev..

[35] Baidhe E., Clementson C.L., Hellevang K., Lin Z. (2025). Comprehensive Analysis of Drying Kinetics, Heat and Mass Transfer, and Thermodynamic Properties in High-Temperature Drying of High-Moisture Corn. J. Food Process Eng..

[36] Yin H., Nie Y., Shen J., Wu W., Dou J., Cheng R., Chen J. (2016). Drying characteristics of diced potato with thin-layer by hot-wind based on Weibull distribution function. Trans. Chin. Soc. Agric. Eng..

[37] Liu Z., Wu W., Shen W., Chen Z., Han F., Xu Y., Wu Z. (2020). A Differential Capacitive Moisture Detection Structure and Method Suitable for Bulk Material Flow. Chinese Patent.

[38] Song J. (2024). Research and Application of Digital Twin Control System for Grain Drying. Master’s Thesis.

